# Machine Learning Analysis of Hydrologic Exchange Flows and Transit Time Distributions in a Large Regulated River

**DOI:** 10.3389/frai.2021.648071

**Published:** 2021-04-15

**Authors:** Huiying Ren, Xuehang Song, Yilin Fang, Z. Jason Hou, Timothy D. Scheibe

**Affiliations:** ^1^Energy and Environment Directorate, Pacific Northwest National Laboratory, Richland, WA, United States; ^2^Earth and Biological Sciences Directorate, Pacific Northwest National Laboratory, Richland, WA, United States

**Keywords:** machine learning, random forest, extreme gradient boosting, spatial heterogeneity, transit time, hydrologic exchange flows

## Abstract

Hydrologic exchange between river channels and adjacent subsurface environments is a key process that influences water quality and ecosystem function in river corridors. High-resolution numerical models were often used to resolve the spatial and temporal variations of exchange flows, which are computationally expensive. In this study, we adopt Random Forest (RF) and Extreme Gradient Boosting (XGB) approaches for deriving reduced order models of hydrologic exchange flows and associated transit time distributions, with integrated field observations (e.g., bathymetry) and hydrodynamic simulation data (e.g., river velocity, depth). The setup allows an improved understanding of the influences of various physical, spatial, and temporal factors on the hydrologic exchange flows and transit times. The predictors also contain those derived using hybrid clustering, leveraging our previous work on river corridor system hydromorphic classification. The machine learning-based predictive models are developed and validated along the Columbia River Corridor, and the results show that the top parameters are the thickness of the top geological formation layer, the flow regime, river velocity, and river depth; the RF and XGB models can achieve 70% to 80% accuracy and therefore are effective alternatives to the computational demanding numerical models of exchange flows and transit time distributions. Each machine learning model with its favorable configuration and setup have been evaluated. The transferability of the models to other river reaches and larger scales, which mostly depends on data availability, is also discussed.

## Introduction

Hydrologic exchange flows (HEFs) are the dynamic two-way exchanges of surface and subsurface waters and constituent substances (e.g., dissolved solutes) between a flowing river channel and the surrounding sediments (Harvey, 2016). In particular, we consider HEFs to represent water that leaves the surface channel, moves through the subsurface environment for some time and distance, then re-emerges into the surface channel. Driven by spatially and temporally varying pressures across the riverbed, HEFs lead to the exposure of surface water constituents to mineral surfaces and microbiological agents that reside in the subsurface environment. The subsurface transit time determines whether kinetically controlled microbial reactions have sufficient time to proceed to completion. These processes promote biogeochemical reactions in the hyporheic zone that represent a potential hotspot of nutrient and contaminant transformations. For example, studies have shown that up to 96% of aerobic respiration in river corridors is associated with sediments exposed to river water through hydrologic exchange (Naegeli and Uehlinger, 1997).

Quantification of HEFs and transit time distributions has been an emphasis of previous river corridor studies (Boano et al., 2014; Cardenas, 2015). HEFs and transit times are determined by the complex interaction among multiple physical features and processes including river morphology (Boano et al., 2007; Cardenas and Wilson, 2007; Tonina and Buffington, 2007; Cardenas, 2008; Stonedahl et al., 2013), sediment and aquifer hydraulic properties such as permeability with spatial heterogeneity (Cardenas et al., 2004; Salehin et al., 2004; Sawyer, 2015; Liu et al., 2020; Rajabi et al., 2020), and river flow variations caused by both natural factors (e.g., floods, tides) (Larsen et al., 2014; Musial et al., 2016) and human factors (e.g., dam operations) (Arntzen et al., 2006; Song et al., 2018). Many previous studies have evaluated these factors individually, or in the context of simplified system representations. More recently, computational capabilities have advanced to allow simulation of coupled surface-subsurface flows in three-dimensions at relatively large scales and with simultaneous resolution of many of these features (Shuai et al., 2019; Fang et al., 2020).

However, three-dimensional (3D) mechanistic simulation of HEFs and transit time distributions (TTDs) remains computationally intensive, especially for large river corridors, and is not feasible for application at large watershed to basin scales. Alternatively, data-driven machine learning (ML) methods can be helpful in improving the capacity of the 3D mechanistic simulation and scaling up to the large domains, by capturing and quantifying the complex correlations between multivariate model inputs and outputs that are descriptive of the underlying physical system. Previous studies have proved that ML models can achieve high prediction on accuracy in hydrologic applications (Hsu et al., 1995; Tesoriero et al., 2017; Barzegar et al., 2018; Mo et al., 2019; Nearing et al., 2020). In this study, we adopted two ML models, Random Forest (RF) and Extreme Gradient Boosting (XGB), given their competitive capabilities to deal with system high-dimensionality, nonlinearity, mixed numerical and categorical variables, highly correlated predictor variables, as well as overfitting, and they have been proven to be superior to traditional ML methods in various case studies (Prieto et al., 2019; Yan et al., 2019; Li et al., 2020; Tavares da Costa et al., 2020; Xenochristou et al., 2020). Bagging/boosting techniques have been used to get ensemble learners in which each individual member of the ensemble is trained using a different training data set subsampled randomly in both rows and columns from the full training data set. This feature selection approach reduced spurious impacts of multicollinearity and has been shown to improve the stability, reliability, and the accuracy of the model even in the presence of highly correlated input variables (predictors) (Strobl et al., 2008; Dormann et al., 2013; Tomaschek et al., 2018). Based on tree-based ensemble learning, RF and XGB offer essential improvements and robustness over single learning algorithm by constructing an ensemble of base and relatively weaker learners to reduce bias and overfitting. Well-trained ML models provide a means to construct models of HEFs and TTDs that can be extrapolated to large domains, with algorithms trained based on 3D mechanistic models of selected representative smaller domains. In order to allow for further explain ability and interpretability of data-driven ML models, tree-based methods are among top choices because they can provide insights into which variables exert the greatest controls on HEFs and TTDs with quantitative measures.

This paper evaluates the feasibility of such an approach using data and models from the Hanford Reach of the Columbia River in the state of Washington, USA. Our study area is the Hanford Reach located in the Columbia River Basin. A 3D groundwater flow and transport model was built using PFLOTRAN-based (Hammond et al., 2014) for the Hanford 100H area under homogenous and facies-based heterogeneous (Hou et al., 2019) riverbed conductance scenarios. The predicted transient velocity field from PFLOTRAN, driven by variable riverbed boundary pressures derived from a 2D river hydrodynamics model, has been used as input to a particle tracking model to obtain exchange fluxes and transit time distributions. The resulting model outputs (HEFs and TTDs), together with a suite of physical variables derived from both observational data and model products, comprise a high-dimensional numerical data set of mixed data types, which is used to develop ensemble tree-based ML models (RF and XGB). The results are evaluated to determine the ML model's predictive power and identify those variables that most strongly influence HEFs and TTDs in the model system. And the favorable condition for applying ML model has been discussed.

## Materials and Methods

### Study Site

**Figure 1:**
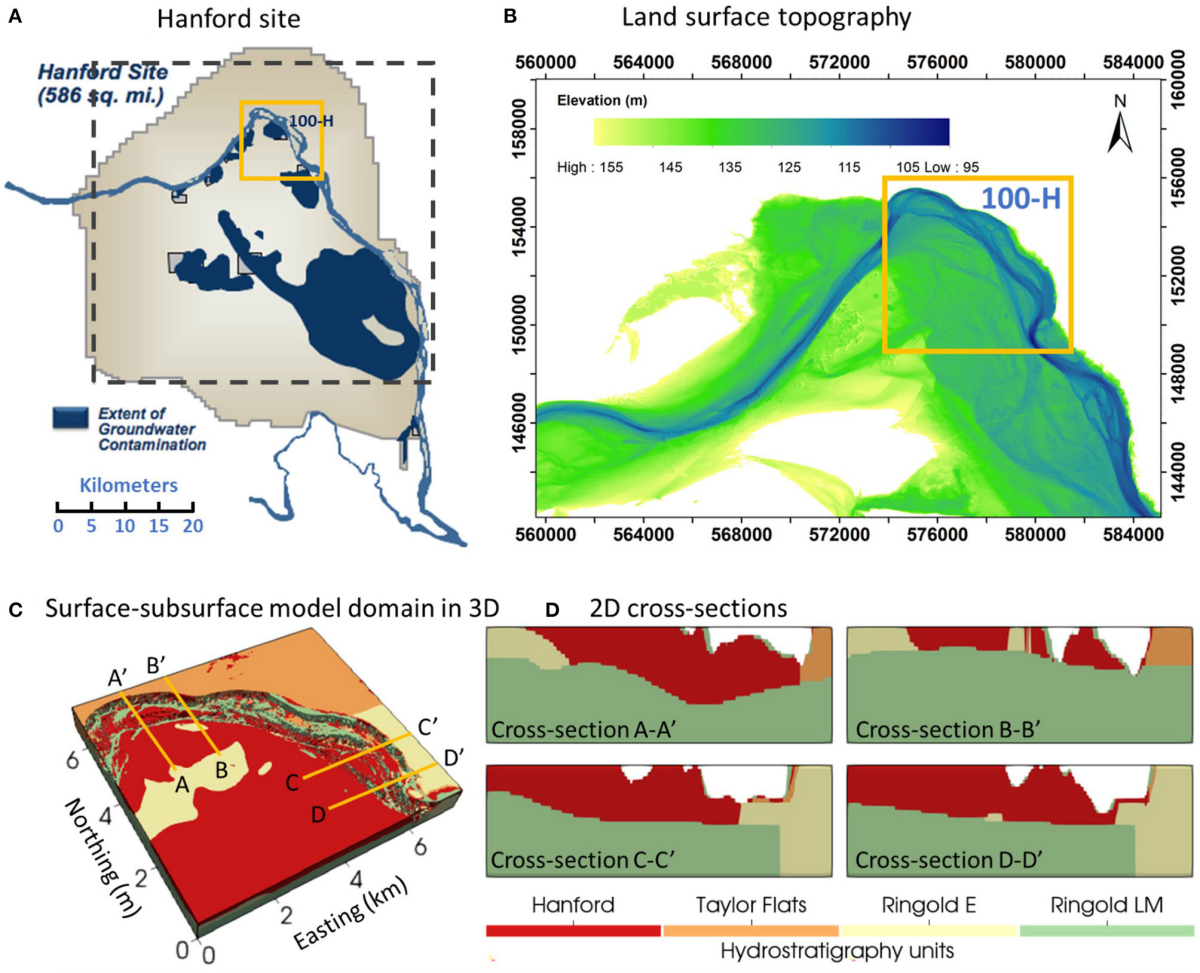
**(A)** Map of Hanford site; **(B)** Land surface topography of the model domain; **(C)** 3D representation of the model domain with various geologic units; **(D)** 2D cross-sections of the model domain. Locations of the cross-sections in **(D)** are as indicated in **(C)**.

The study site is the Hanford Reach, which is a section of the Columbia River located in southeastern Washington State, USA, as shown in the upper right panel of Figure 1. The reach extends ~85 km from the tailrace of Priest Rapids Dam to the north end of the city of Richland. River discharge in the Hanford Reach is regulated by a series of upstream hydroelectric dams. The river stage at the study site fluctuates up to 2~3 m annually and ~0.5 m daily because of annual snow melting events and power generation schedules (Arntzen et al., 2006). The model domain is located at 100H area, marked as the yellow box in Figures 1A,B. The riverbed sediment in the Hanford Reach is predominantly coarse gravel ranging in size from granules to boulders with fine sediments infilling gaps between large clasts (Rakowski et al., 2006). The land surface in the area is relatively flat, as shown in the land surface topography map (Figure 1B). The unconfined aquifer in the river corridor consists of two major geologic units: (1) the upper coarse-grained Hanford Formation and (2) the lower less-permeable Ringold Formation (Thorne et al., 2006). The Ringold Formation in the 100H area can be further divided into three textural subunits, including Ringold Taylor Flats, Ringold E, and Ringold Lower Mud (LM). In the physical system, there is an alluvial layer on the riverbed that is geologically and hydraulically distinct from the underlying surficial aquifer. It is on the order of 1–2 m thick as indicated by geophysical surveys and direct push observations, and generally has lower permeability and porosity than the underlying aquifer, especially where underlain by the highly permeable Hanford formation. Characteristic values of hydraulic conductivity and porosity for each geologic unit are summarized in Table 1. The model domain for 3D groundwater flow and transport model with geologic units is in Figure 1C and the 2D cross-sections at selected locations have been demonstrated in Figure 1D. In our study, the river is fully connected with the surrounding surficial aquifer.

**Table 1:** Hydrogeologic properties of geologic units.

	**Hanford**	**Taylor flats**	**Ringold E**	**Ringold LM**
Horizontal hydraulic conductivity (m/day)	6255.17	0.89	35.68	0.89
Anisotropy ratio	0.1	0.1	0.1	0.1
Porosity	0.20	0.43	0.25	0.43

### Hydromorphic Unit (HU) Classification

**Figure 2:**
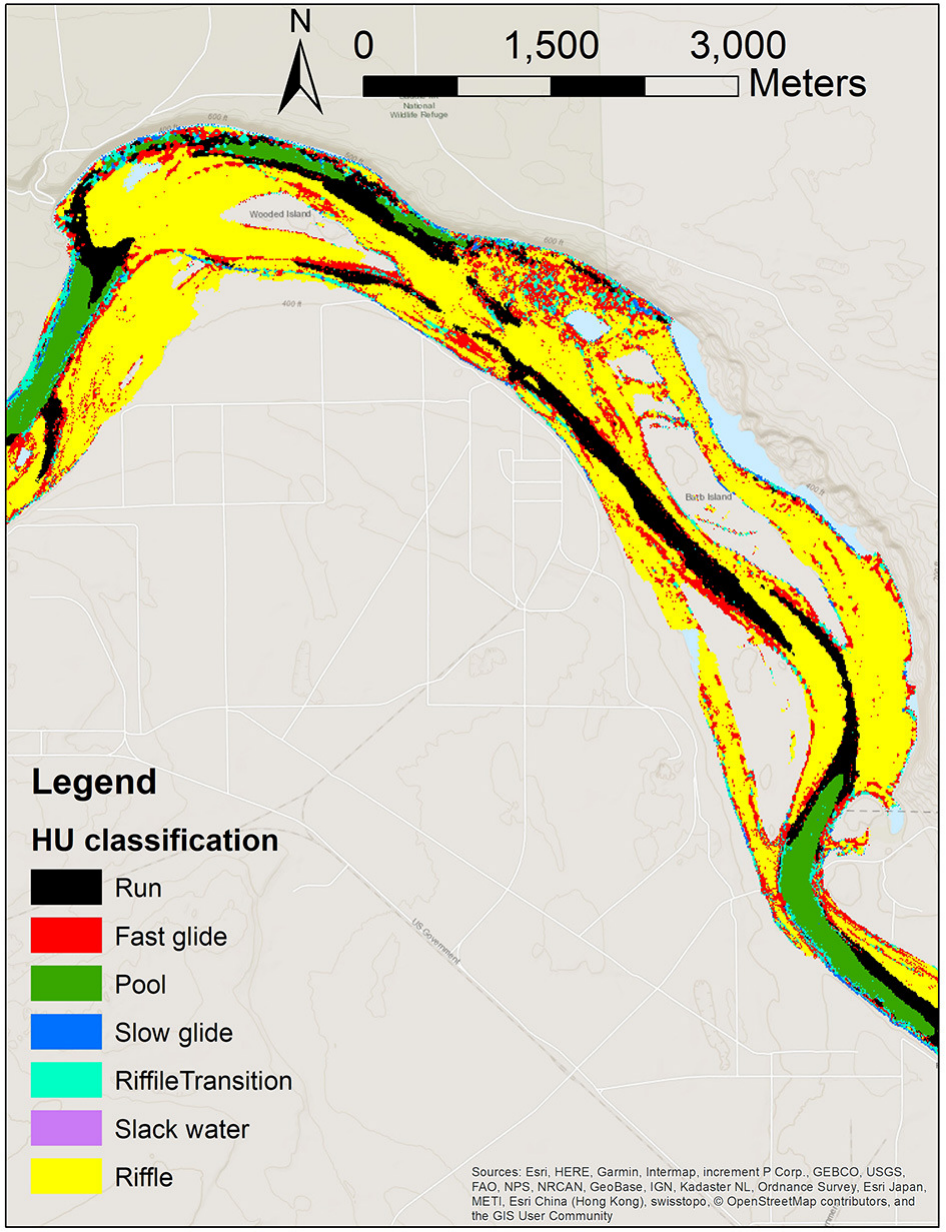
Map of hydromorphic unit (HU) classes in the simulation area.

HEFs are driven in part by local pressure variations associated with interactions of surface flows with riverbed morphology (hydromorphic structure). We hypothesized that the characterization and mapping of hydromorphic features could provide insights into the variability in HEFs and TTDs. In related work being reported elsewhere (Hou et al., 2021), we developed a hydromorphic unit (HU) classification system (Figure 2) that is used here for two purposes: (1) to assign spatially variable riverbed conductance values in PFLOTRAN simulations, and (2) as a categorical indicator variable in the machine learning analysis. Both of these are discussed further below.

The HU classification system is based on k-means clustering applied to bathymetric data and hydrodynamic model outputs, and results in maps of several classes of HUs with associated descriptors as used in standard hydromorphic taxonomies [e.g., (Wheaton et al., 2015)], such as “run,” “fast glide,” “pool,” “slow glide,” and others. For example, pools are characterized by large depth, low to intermediate velocity, low riverbed slope, and concave zones; while runs have intermediate depth, intermediate to large velocity, low riverbed slope, and can be concave, flat, or convex.

### Coupled Surface-Subsurface Flow Model

A highly-resolved, 3D coupled surface-subsurface flow and transport model was previously developed for our study site (Fang et al., 2020). Here, we use the outputs of that complex model to train machine learning models of system behavior that can be used as surrogates for the computationally intensive and data-demanding physics-based model. Here we provide a brief summary of the methods used in the 3D model, and refer the reader to (Fang et al., 2020) for additional details.

Fang et al. (2020) used the state-of-the-art massively parallel subsurface flow and reactive transport code PFLOTRAN to simulate the dynamic hydrologic exchange fluxes and subsurface velocity fields. PFLOTRAN solves a system of nonlinear partial differential equations to describe multiphase, multicomponent, and multiscale reactive flow and transport in porous materials (Hammond et al., 2014). The 3D Richards' Equation for variably saturated flow was solved for this study which can represent both fully and partially saturated conditions. Therefore, it can handle situations such as fluctuating water tables, seepage faces, and so forth that occur even in this fully connected river-aquifer system. The constitutive relationships used for saturation and relative permeability were the van Genuchten (1980) and Burdine (1953) models, respectively. The simulation period is from 2013 to 2015, which covers two normal flow years and a low flow year in 2015.

As described in Fang et al. (2020), the 2012 results of a coarser reach scale simulation in Shuai et al. (2019) were interpolated to prescribe the initial and groundwater boundary conditions of the modeled domain. The bottom boundary of the domain was defined as no flow condition as it was constrained by the low permeable Ringold units. The top boundary was also set as no-flow as the surface recharge is negligible in the semiarid climate zone (Rockhold et al., 1995). The inland boundaries of the domain were prescribed as hydrostatic conditions, using transient pressures interpolated from the reach scale simulation in Shuai et al. (2019).

The transient river boundary condition was derived from hourly river stages simulated using the Modular Aquatic Simulation System in two dimensions (MASS2) simulator (Perkins and Richmond, 2007), which is a 2D depth-averaged river model. To represent a low permeable thin alluvium layer at the sediment-water interface, a conductance type boundary condition was imposed on each grid cell of the riverbed. The conductance type condition is similar to a seepage face, which dampens the effect of river stage fluctuations (Hammond and Lichtner, 2010). For most of the simulations reported here, a value of 1.0 × 10^−12^ m was applied homogenously across the entire riverbed surface. To test the potential impacts of riverbed heterogeneity on the model predictions, we also considered one case in which different values of conductance were applied as a function of the HU observed at each grid cell location, based on the association of HUs with riverbed substrate size maps (Ren et al., 2020). Table 2 lists the values of the conductance coefficient assigned to each HU for homogeneous and heterogeneous cases. The simulation domain has structured grids without adapted meshing to changing slopes. Unstructured grids may better present geological features following their boundaries and are beneficial for mechanistic modeling in complex domains (Käser et al., 2014; Su et al., 2020; Manzoor et al., 2021). But structured grids have been commonly chosen for simulating large domains given their sufficiency and computational affordability.

**Table 2:** Conductance coefficients (in m) of riverbed HU.

	**HU 1**	**HU 2**	**HU 3**	**HU 4**	**HU 5**	**HU 6**	**HU 7**
Homogenous case	1.00 × 10^−12^	1.00 × 10^−12^	1.00 × 10^−12^	1.00 × 10^−12^	1.00 × 10^−12^	1.00 × 10^−12^	1.00 × 10^−12^
Heterogenous	1.37 × 10^−11^	4.89 × 10^−13^	1.15 × 10^−11^	1.11 × 10^−13^	4.80 × 10^−13^	3.49 × 10^−14^	5.29 × 10^−13^

The maximum time step of the simulation is 1 h. The wall-clock time for one simulation was 20 h or more (depending on solution convergence) using 1,536 process cores on the Cascade high performance computing (HPC) cluster at the Environmental Molecular Sciences Laboratory (EMSL). 28.76 terabytes of output in hdf5 format for each simulation were saved on disc for post processing.

### Particle Tracking Model

We used forward particle tracking to estimate the transit time distribution (TTD) of surface water through the river corridor subsurface aquifer by tracking the movement of water particles. A classical semi-analytical particle-tracking algorithm (Pollock, 1994) was adopted in this study by using the velocity outputs from PFLOTRAN as inputs; see details in (Song et al., 2020).

One hundred thousand (10^5^) numerical particles were randomly released along the river boundary of the PFLOTRAN model domain at each of 1,000 time points randomly selected between October 2013 and September 2014. The HEFs and transit times of all particles were recorded for use in the subsequent analysis. The exchange flux rate of each particle is defined as the Darcy velocity modeled by PFLOTRAN at the location and time of particle release. The transit time of each particle is defined as the time elapsed from entering the riverbed to exiting through the aquifer. Noted the terms “transit time” and “residence time” have in some studies been used interchangeably [e.g., (Cardenas et al., 2004; Stonedahl et al., 2010; Trauth et al., 2013)], here we distinguish between transit time and residence time, with the residence time being the elapsed time since a subsurface particle of river water left the surface channel, and the transit time being the residence time at the point of return to the surface channel [e.g., (Schmadel et al., 2017)]. This particle tracking exercise provided a large dataset that includes exchange flux rate and transit times of 100 million particles. Each particle was weighted according to the local exchange flux rate to reflect the fact that more water enters in zones of higher exchange. Transit time distributions (TTDs) are then defined as the flux-weighted probability density functions (PDFs) of the time elapsed between river water entering and leaving the river corridor. We performed convergence tests with sequentially increasing numbers of particles to determine that the number of released particles was sufficient to provide stable estimates of transit time and exchange flux rate distributions.

### Predictor and Response Variables for Machine Learning

A central objective of this research is to apply machine learning models to relate simulated HEFs and TTDs from high-resolution mechanistic simulations to variables that are relatively easily observed or measured, both to provide alternative (surrogate) predictors and to gain understanding of the relative influences of a range of variables on HEFs and TTDs.

Here we define the set of input variables used as predictors, and the summary metrics of HEFs and TTDs used as response variables (predictions), in the machine learning training and prediction process described below. Table 3 provides a summary of the input/predictor and output/response variables, and the following paragraphs provide additional details on their definitions. The inputs (predictors) can be either continuous or categorical variables, and the set of inputs comprises both observed (measured) variables and simulated (modeled) variables from the high-fidelity mechanistic simulations described above. The response (output) variables in this case are both continuous variables and comprise summary metrics of HEFs and TTDs computed from the outputs of the high-fidelity mechanistic simulations.

**Table 3:** Summary table of the ML model predictors and responses for the base configuration.

		**Bathymetric attributes**	**Hydrodynamic attributes**	**Flow regimes**	**Geomorphologic attributes**
Predictors	Continuous (real number) variables	Elevation, slope, aspect ratio, curvature	First four statistical moments (over time) of water surface elevation, velocity, shear stress, and water depth	Separate analysis performed on data subsets from each of the four flow periods (P1, P2, P3, P4)	None
	Categorical (discrete) variables	Concavity	None	Flow period (P1, P2, P3, P4) as categorical variable for the case with all flow periods combined	HU type, Hanford Formation thickness class
Responses	Mean flux rate and transit time (averaged over all particles emplaced on each MASS2 grid cell).				

*All variables are defined at each of the MASS2 grid cells*.

#### Bathymetric and Hydrodynamic Attributes

Riverbed bathymetry and hydrodynamic features are thought to be strongly related to HEFs and TTDs, and thus form one important class of potential predictor variables.

For this study, riverbed digital elevation data were available from prior analyses that combined LiDAR and field bathymetric surveys to construct a complete bathymetric surface on a grid resolution of 1 m (Coleman et al., 2010). In addition to the raw elevation (m above mean sea level) of the riverbed at a grid cell, four derivative metrics of riverbed bathymetry were extracted from the gridded elevation dataset. For each grid cell, the slope and aspect were calculated using available functions in the Environmental Systems Research Institute (ESRI) ArcGIS platform. The slope represents the maximum steepness of the riverbed surface within a grid cell and is calculated as the inverse tangent of the rise divided by the run. The aspect represents the compass direction associated with the steepest slope and is measured clockwise from 0 to 360°, with 0 being azimuth north. For non-sloping (flat) cells, aspect is flagged with a value of “−1.” The curvature (also called convexity or concavity) is the second derivative of the surface elevation (the rate of change of the slope). Positive curvature indicates the surface of the grid cell is convex upward, negative curvature indicates the surface is concave upward, and a value of zero indicates a flat surface. Thus, the curvature variable is a continuous variable while the concavity is categorical (convex upward, concave upward, or flat).

Hydrodynamic variables were also available from previous simulations using the Modular Aquatic Simulation System 2D (MASS2) code (Perkins and Richmond, 2007). MASS2 is a two-dimensional (depth-averaged) hydrodynamic model that uses an orthogonal curvilinear mesh. In the previous work, MASS2 was applied over a river reach ~97 km in length, from Priest Rapids Dam to near the mouth of the Yakima River. The model used ~710,000 computational cells with a nominal resolution of 10 m. MASS2 calibrations were performed using measured water surface elevations at various locations along the Hanford Reach, with mean absolute errors varying from 1 to 12 cm. A transient simulation was performed over a long historical period (1917–2012) for which discharge records are available. Because of a series of upstream dams, flood discharges, and sediment loads have been greatly reduced from natural conditions. Sedimentological studies of our site (Fecht et al., 2004; Fecht and Marceau, 2006; Hou et al., 2019) have determined that the bed topography and surface sediment distribution (mostly gravels and cobbles) are highly stable under the current flow conditions. Therefore the underlying bathymetry was assumed to be constant over the simulation period. Biologically our system is oligotrophic, with insufficient organic matter to drive enough growth of biofilms in the subsurface environment to cause clogging. The primary source of organic matter to the hyporheic zone appears to be particulates from a riverbed surface layer of phototrophs (e.g., algae) penetrating into the riverbed (Stern et al., 2017; Roden et al., 2019), and while we are interested in this process because of its biogeochemical implications, preliminary results indicate that this particulate matter would be of insufficient volume to significantly impact permeability or porosity. Therefore, we assume the riverbed conductance does not vary temporally, although we recognize that in some systems this may be an important consideration (Gianni et al., 2016). Details of model calibration and long-term transient simulation are documented in Niehus et al. (2014). For each MASS2 grid cell, a number of metrics were computed from the simulation outputs including the wet percentage (percent of time the grid cell was submerged during the simulation period) and statistical moments (mean, variance, skewness, and kurtosis) of the water depth, velocity magnitude, riverbed shear stress, and shear velocity. These metrics, together with the bathymetric predictors described above (mapped onto the MASS2 grids) were used as input predictor variables in the subsequent machine learning analyses.

#### Geomorphologic Attributes

Two geomorphologic attributes were used as input predictor variables: (1) the hydromorphic unit (HU) type assigned to each grid cell of the model where particles were placed (see Section Hydromorphic Unit (HU) Classification), and (2) the thickness of the Hanford formation at the location of each grid cell (see Section Study Site). The HU type is hypothesized to be related to large-scale hydrostatic and dynamic pressure gradients that drive HEFs, and the geometry of the highly permeable Hanford formation is expected to strongly influence fluxes and flow path lengths. HU type is inherently a categorical variable, and the Hanford formation thickness was discretized into 28 bins to create a categorical representation as well.

#### Flow Regimes

**Figure 3:**
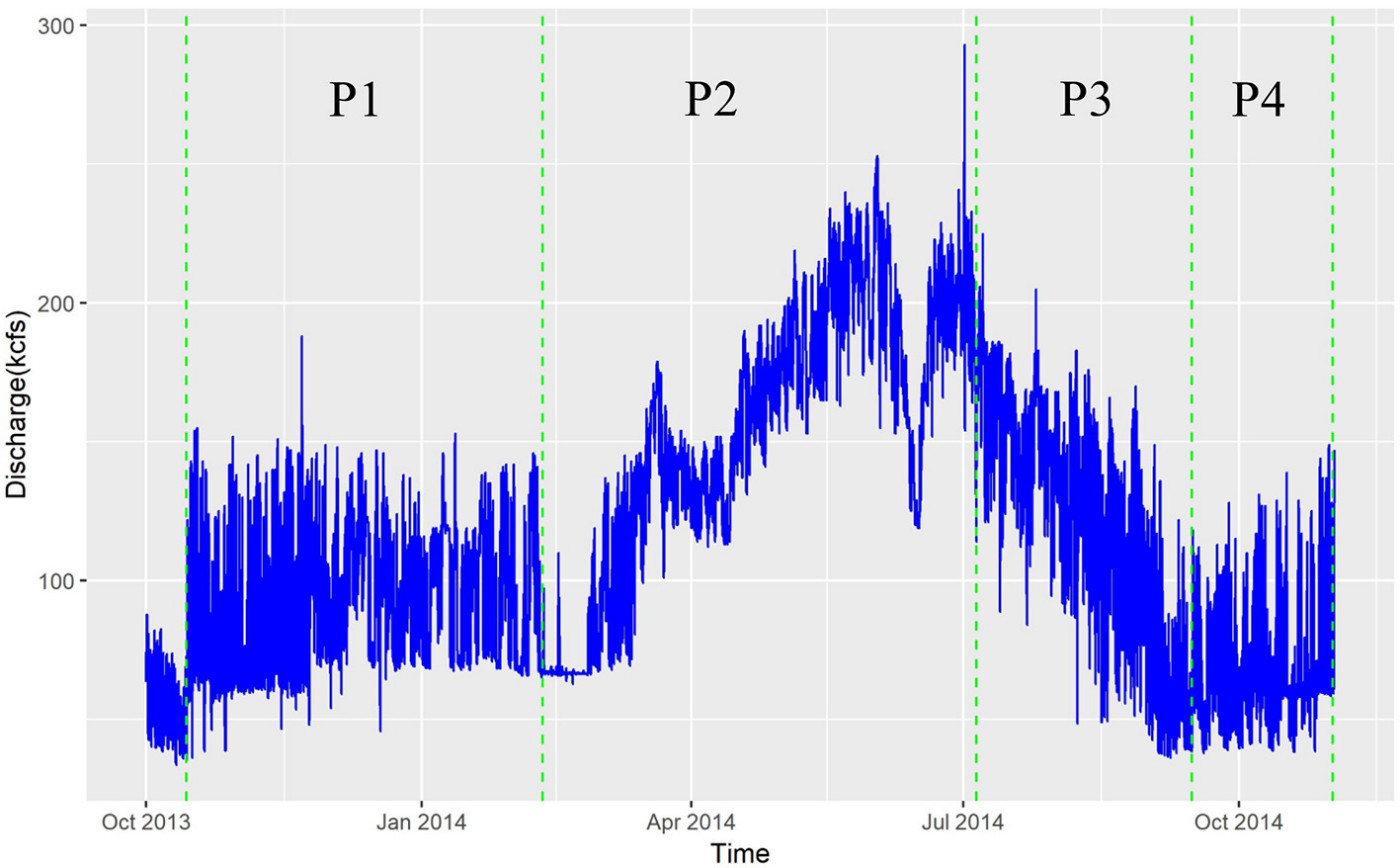
Discharge from U.S. Geological Survey gaging station 12472800, Columbia River below Priest Rapids Dam, WA for the period of approximately water year 2014.

In a highly dynamic flow system such as the Columbia River's Hanford Reach, HEFs, and TTDs are likely to depend strongly on the flow regime. River discharge in the Hanford Reach is regulated by upstream dams and most directly by the Priest Rapid Dam, a low-head hydroelectric facility at the upstream end of the Hanford Reach. Hourly discharge data for Priest Rapids Dam are available from the U.S. Geological Survey gaging station 12,472,800, Columbia River below Priest Rapids Dam, WA. The observed Priest Rapids Dam discharge time series for the 2014 water year, which falls within the time period of the PFLOTRAN groundwater flow simulation, is shown in Figure 3. Four flow regimes were qualitatively defined from this time series: (1) Period 1 (P1) from October 2013 to February 2014, a period with relatively stable moderate flow and a median discharge of 86 kilocubic feet per second (kcfs), (2) Period 2 (P2) from February to July 2014, a period with generally increasing flow and median discharge of 160 kcfs; (3) Period 3 (P3) from July to September 2014, a period with generally decreasing flow and median discharge of 128 kcfs, and (4) Period 4 (P4) from September to November 2014, a period of relatively stable low flow and median discharge of 62 kcfs.

To test the hypothesis that HEFs and TTDs depend on flow regime, machine learning analyses were conducted separately for each of the four flow periods above, as well as for all flow periods combined. For the analysis of the combined data, the flow regimes were included as categorical input predictor variables.

### Principal Component Analysis

It is likely that some or many of the variables defined above as input predictors are linearly correlated with each other. For example, the HU class is defined based on some bathymetric and hydrodynamic variables used as input predictors. Therefore, it may be possible to reduce the dimensionality of the input predictor variable set, decreasing the computational demand of training and executing machine learning models without losing significant predictive power. Here we have explored this possibility using Principal Component Analysis (PCA).

PCA seeks to replace *p* (more or less correlated) variables by *k* < *p* uncorrelated linear combinations (projections) of the original variables. Those *k* principal components are ranked by importance through their explained variance, and each variable contributes with varying degree to each component. It is helpful to address potential multi-collinearity issues, but we note that PCA does not take into account the potential multivariate nature of the data structure (e.g., higher order interaction between variables). On the other hand, PCA and clustering analysis can at least provide some guidance on the actual dimensionality of the predictor matrix before applying more complicated machine learning models to help with physical interpretability of results.

The other use of PCA is that it can extract cross-dependence structure among the predictors, therefore, in practice, if only a subset of predictors are available, one can tell if all the major variabilities or behaviors can be captured with existing data and therefore provide guidance on future data acquisition needs or evaluating the feasibility and transferability of ML models with subset data.

### Random Forest

An ensemble tree-based machine learning approach, Random Forest (RF), was used here to address the high dimensionality of the predictor variables and potential nonlinear relationships among HEFs, TTDs, and the predictor variables. RF uses a collection (ensemble) of tree predictors [*h*(***x***, **Θ**_*k*_), *k* = 1, 2, 3, …] where the **Θ**_*k*_are independent identically distributed random vectors and ***x***is the input vector (Breiman, 2001). To grow a RF, each tree is grown using a randomly generated subset of the full training data set by resampling randomly with replacement from the original (full) training data set using a bootstrap aggregating (bagging) technique.

Each tree comprises a series of decision nodes or branching points at which the tree assigns the decision based on the observations in its subset of the training data (Pal, 2005; Rodriguez-Galiano et al., 2012). The final prediction is made by averaging the predictions from all the individual regression trees in the ensemble so generated. User-defined parameters are required, including the number of trees in the ensemble and the number of predictive variables used to split the nodes. Previous studies have shown that bagging methods like RF are not sensitive to outliers or noisy data (Briem et al., 2002; Chan and Paelinckx, 2008). For each individual tree, those input samples that were not included in the randomly generated subset of training data are tracked as “out-of-bag” (OOB) data in the bootstrap sample. The proportion of misclassifications over all OOB data sets is called the OOB error and is an unbiased estimator of the generalization error (Breiman, 2001; Peters et al., 2007). The convergence of the generalization error provides a means to estimate the required number of trees. An advantage of RF is that it allows individual trees to grow to the maximum possible depth using a given combination of input variables (Mingers, 1989; Pal, 2005), and it also provides measures of the relative importance of the features in the predictions.

RF is well suited to analysis of high-dimensional data sets including highly correlated input features, and it has been successfully applied to analyses of soil microbial communities, remote sensing classifiers, water resources data, and many earth science problems (Heung et al., 2014; Naghibi et al., 2017; Tesoriero et al., 2017).

### Extreme Gradient Boosting

Although RF models are known to generate effective predictions while minimizing problems with overfitting, it is often useful to compare multiple machine learning approaches on a given data set. Here we use, in addition to RF, another widely accepted ensemble machine learning model, gradient boosting model (GBM) (Friedman et al., 2000). Like RF, GBM is tree-based, but the primary difference is that RF builds each tree independently while boosting-based GBM builds one tree at a time, with each tree learning from and improving upon the previous one by minimize the error which is also known as weighted tree-growing algorithm. Each tree is growing with the modified version of the original dataset based on previous trees built. While RF combines results over the ensemble at the end of growing all trees, GBM combines results throughout the tree-building process. Relative to RF, GBM is more computationally intensive and is more sensitive to noise in the training data set. Extreme Gradient Boosting (XGB) (Chen et al., 2015) is adopted which is built on GBM and designed to provide a scalable and efficient implementation. User-defined parameters that can be used to tune the XGB model are the number of trees, the boosting learning rate, the number of splits in each tree, and the subsample ratio of columns when constructing each tree.

This study was implemented using the open-source statistical software R (R Core Team, 2018), within which the R implementation of the H2O package (Aiello et al., 2016), a scalable and distributed platform, was used for RF and XGB model development, validation, and prediction.

## Summary of Machine Learning Analyses Performed

This study used multiple different input variable sets and two different machine learning models (RF and XGB) to generate a total of 15 alternative analyses. Over the entire simulation domain, both predictors and responses are defined at approximately 55,000 MASS2 simulation grid cells (5–10 m spatial resolution). For development of the ML models with optimal model configuration, this full data set was randomly separated into training data (70%) and validation data (15%). The remaining 15% of the data served as a third independent data set to test the ML model performance. These three separate components of the data set lead to well-tuned model hyperparameters and an unbiased model estimation for both RF and XGB models.

To clarify the different cases considered, we provide here a brief summary overview of the analysis workflow, as follows:

The base configuration (C0), with input and output variables as defined in Table 3 and homogeneous riverbed conductance, resulted in 10 analysis cases [5 groupings of data, 4 for the temporal subsets based on flow regime plus 1 for all flows combined, each analyzed using 2 machine learning models (RF and XGB)].

Three additional variable configurations were also considered:

C1: Same as the base configuration, but only considering the combined flow regime and adding the map coordinates of each grid cell as predictor variables. Two analysis cases (RF and XGB).

C2: Reduced set of input variables from the base case, with the subset of variables selected using PCA analysis. Only the combined flow regime was considered. Two analysis cases (RF and XGB).

C3: Same as the base configuration, but only considering the combined flow regime and using PFLOTRAN outputs based on heterogeneous conductance values (see Table 2). One analysis case (XGB only).

## Results

We first present some overview results of the numerical modeling exercise under the different flow regimes considered. We then present ML model performance (in terms of prediction accuracy on the testing data set) for analyses of the base configuration with different treatments of flow regime. XGB and RF results can be compared to determine favorable conditions for applying either or both. For the base configuration, we present a comparison between XGB and RF models in terms of the most influential input variables and goodness of fit. Lastly, we present results for the three additional configurations defined above.

### Impact of Flow Regime on Flux and Transit Time

**Figure 4:**
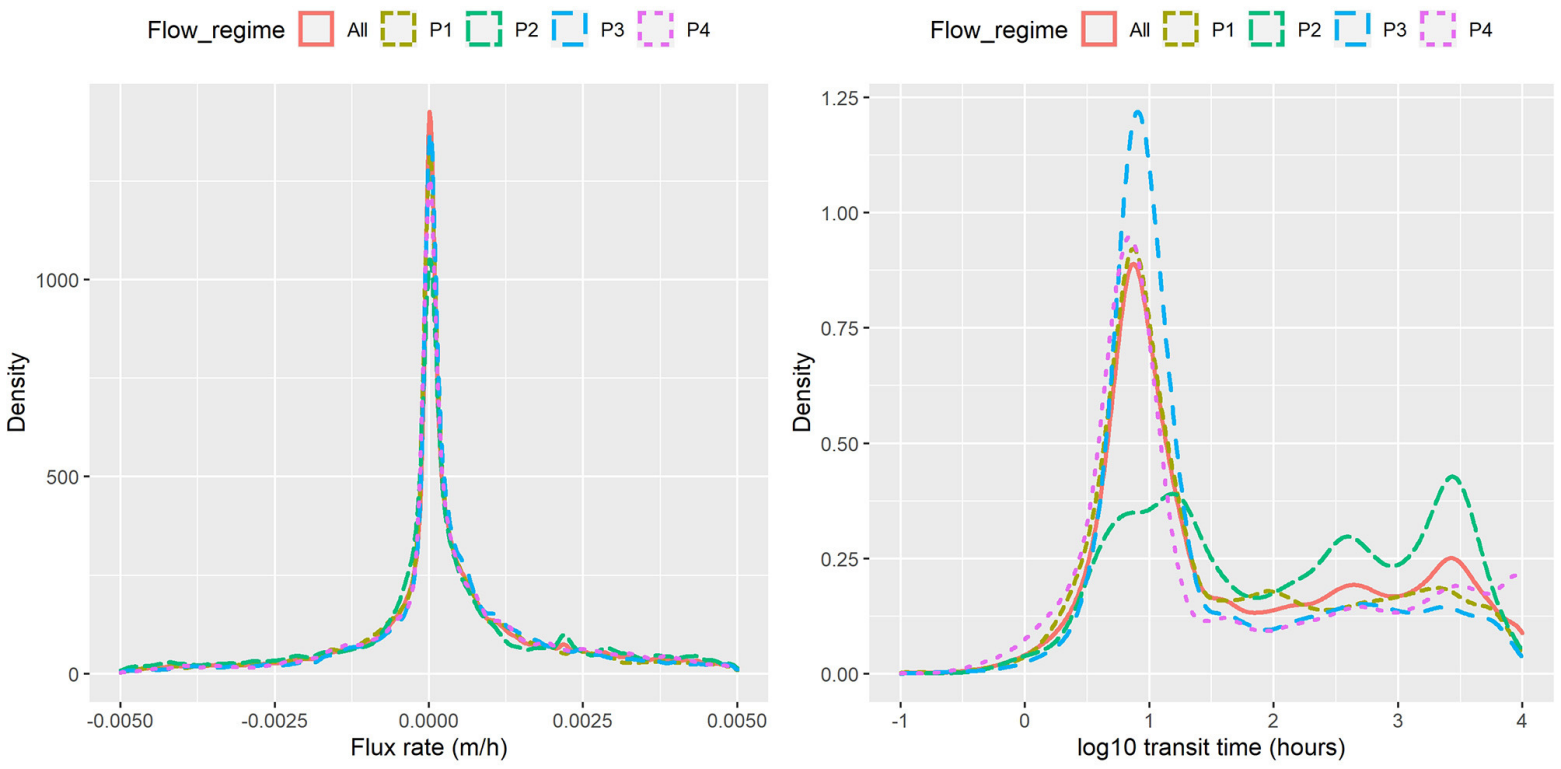
Flux rate and transit time obtained from particle tracking under different flow regimes.

The probability density functions (PDF) of exchange flux (m/h) and transit time (hour, plotted on log scale) under the four flow regimes of Figure 3 (P1, P2, P3, P4) as well as all flows combined (All) are shown in Figure 4 for the base configuration (C0). Within this configuration, model outputs were grouped under the four flow regimes and considered as a whole. The increasing flow regime (P2) is clearly distinctive from the other regimes, with slightly larger magnitudes of exchange fluxes (HEFs) and significantly longer transit times. The other three regimes have similar distributions of HEFs and transit times, although the low steady flow regimes (P4) appears to have the shortest transit times among the four.

**Figure 5:**
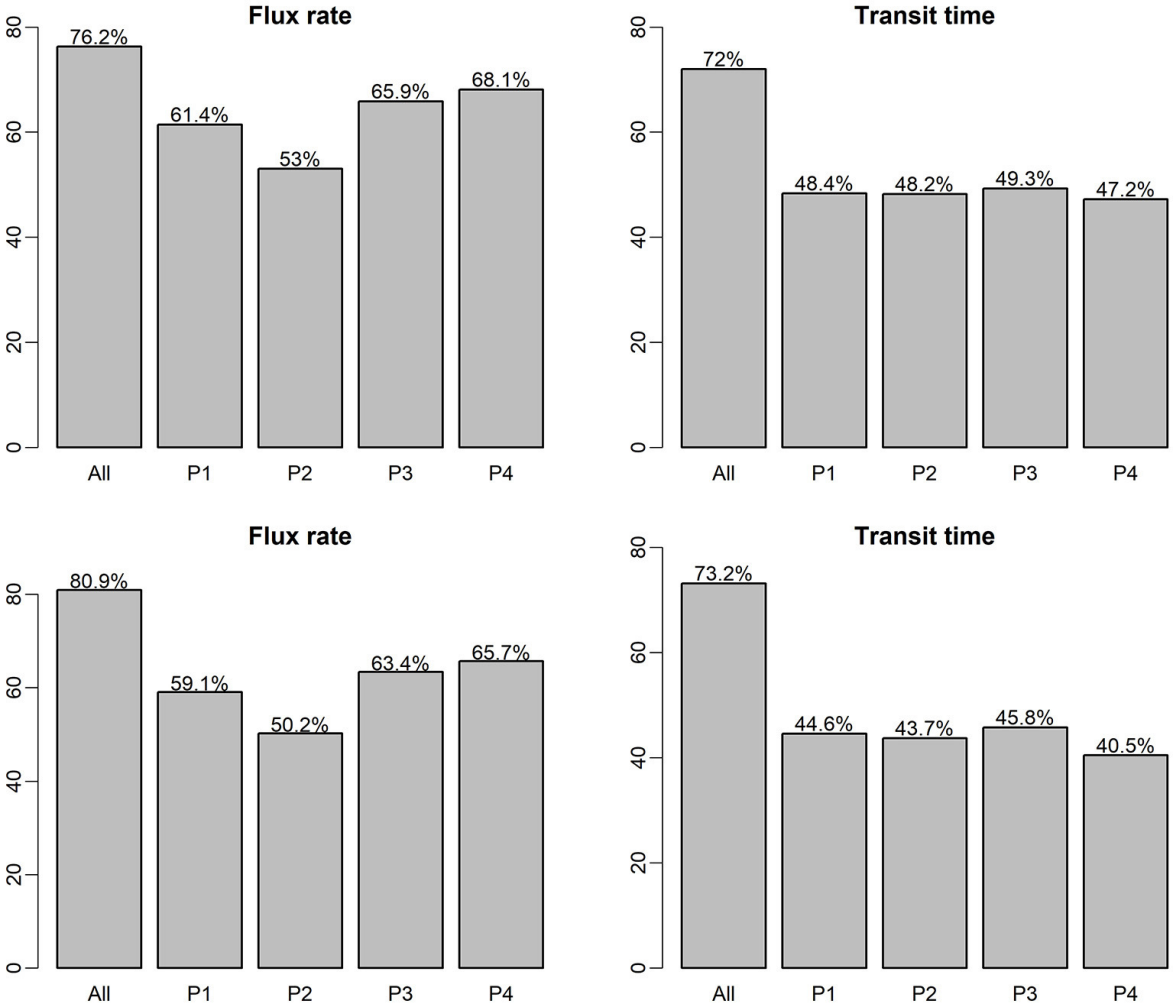
ML model prediction *R*^2^ on testing dataset for single-ML and flow regime-specific ML models, the top panel is RF model, the bottom panel is based on XGB model.

Figure 5 shows the RF and XGB model prediction scores (R^2^ of predictions vs. observations for the testing data set) for the two predictor variables (flux and transit time), for each of the flow regimes (P1, P2, P3, P4, and all flows combined), which the top panel is based on RF model and the bottom panel is based on XGB model. The plot indicates that for both RF and XGB separation of the dataset into flow regimes for individual ML analyses did not improve the performance. The use of flow regimes as categorical indicators in the case of all flows combined (“All” in Figure 5) leads to improved predictions over analyses using individual flow periods. RF and XBG models performed similarly in all these cases. In general, without the temporal component in each flow regime-specific ML model, prediction of HEFs is more accurate than that of TTDs. Prediction of HEFs under the relatively stable low flow regime (P4) is the best of the four flow regimes, but still poorer than that for the all flows combined. Flow regime P2 has the lowest R^2^ for HEF prediction, indicating that fluxes are more difficult to predict under increasing flows. This suggests that the temporal resolution can be relatively coarser for model simulation and field measurement under stable flows but need to be more highly resolved under an increasing flow regime.

### Variable Importance—Base Configuration With Combined Flows

**Figure 6:**
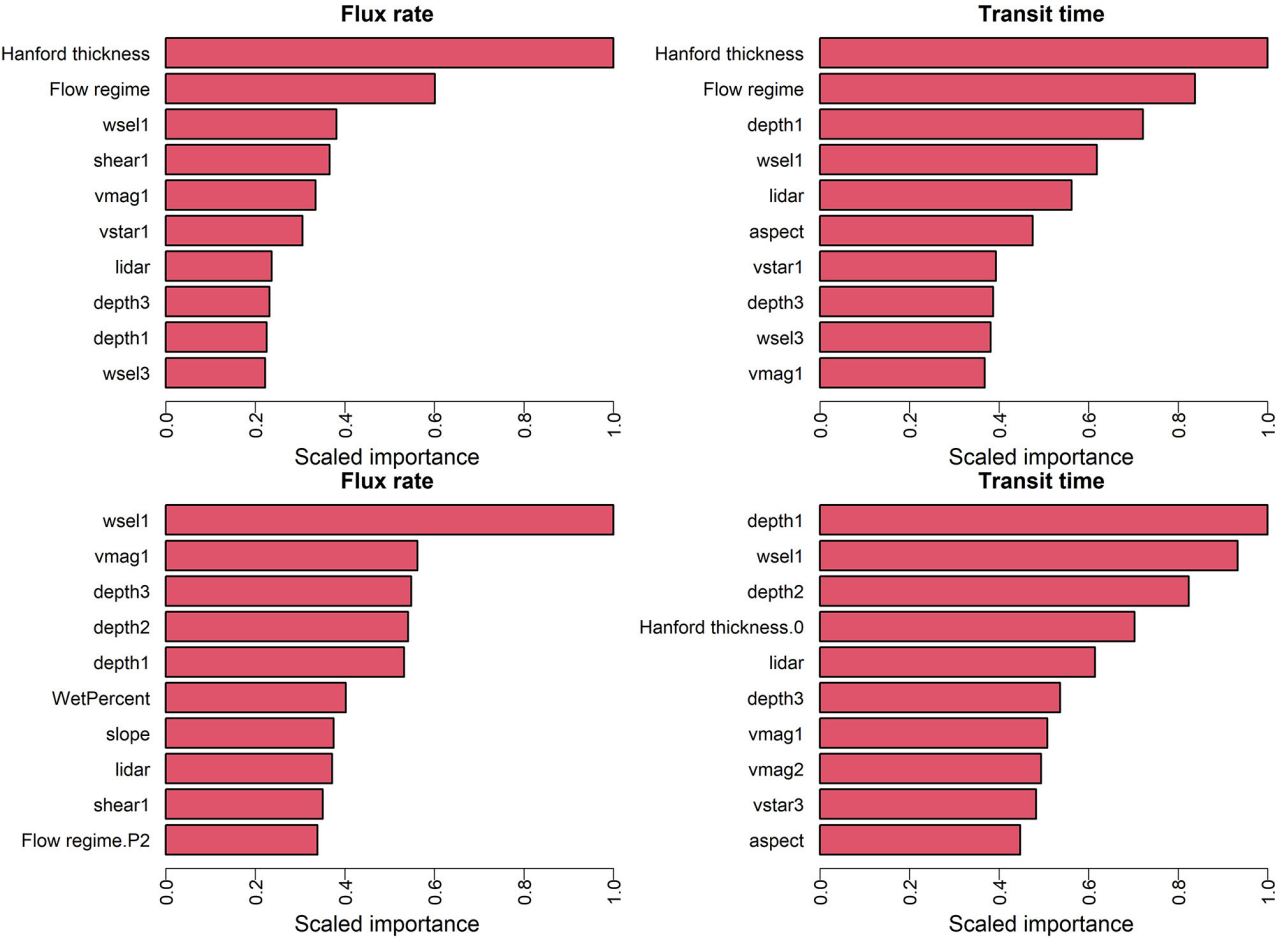
Top 10 variables in terms of scaled feature importance for the optimally trained ML model trained, for prediction of flux (left) and transit time (right). The top panel is based on RF models and the bottom panel is based on XGB models.

The variable importance [or feature importance; (Saarela and Jauhiainen, 2021)] represents the relative influence of each input variable in the model predictions and can be estimated using both RF and XGB models. Because the previous results indicate the best performance is obtained using the combined flow data set (with flow regime as a categorical input variable), we focus here on the base configuration (C0) with all flows combined. Figure 6 shows the 10 most influential input variables with scaled importance based on the ML model for flux rate (left panel) and transit time (right panel) where the top panel is based on RF models and the bottom panel is based on XGB models. For both RF models, the Hanford Formation thickness (geomorphologic attribute), and the flow regime are the most significant variables. For flux prediction, the remainder of the top 10 are predominantly hydrodynamic attributes such as the water surface elevation, shear stress and velocity. Both hydrodynamic and bathymetric attributes (e.g., aspect, elevation) strongly impact the prediction of transit times. In both cases, hydrodynamic variables are dominated by the mean (first moment), while the skewness (third moment) of depth, and water surface elevation are ranked lower.

The bottom panel of Figure 6 shows the 10 most influential predictors with scaled importance based on the XBG model for flux rate (left panel) and transit time (right panel). By comparison to the RF results in the top panel of Figure 6, it is clear that the ranking of variable importance varies significantly between the two machine learning models. Notably, the flow regime and Hanford Formation thickness, which dominated variable importance for RF, are nearly absent from the top ten list for the XGB model. For prediction of flux using the XGB model, the top six variables belong to the hydrodynamic attributes including the mean (first moment) of water surface elevation and velocity and the first three moments of water depth. Bathymetric attributes (slope and elevation) show up lower in the list, and the flow regime (specifically P2) contributes as the tenth most important parameter. The Hanford Formation thickness shows up only as the fourth most influential variable in prediction of transit times, and most variables important to transit time prediction are hydrodynamic or bathymetric.

Feature importance measures are often used in efforts to increase the explainability of machine learning results. Here, those key variables identified as most important differ depending on the ML technique used, and while they represent physical attributes or processes that are well known to influence HEFs and TTDs, it is not yet clear to what degree these measures are indicative of actual physical controls. While understanding feature importance remains an active research area in explainable AI, it has been suggested that combining multiple ML methods is necessary to increase interpretability of the predictions (Saarela and Jauhiainen, 2021). In this work, the importance of several input variables was identified by both ML models and is consistent with understanding from previous studies, including river bathymetry features (Cardenas and Wilson, 2007; Tonina and Buffington, 2007; Stonedahl et al., 2013), flow regime (Sawyer et al., 2009; Larsen et al., 2014; Musial et al., 2016) and aquifer properties (represented by Hanford thickness) (Salehin et al., 2004; Shuai et al., 2019).

## Further Analysis of Model Predictions—Base Configuration With All Flows Combined

**Figure 7:**
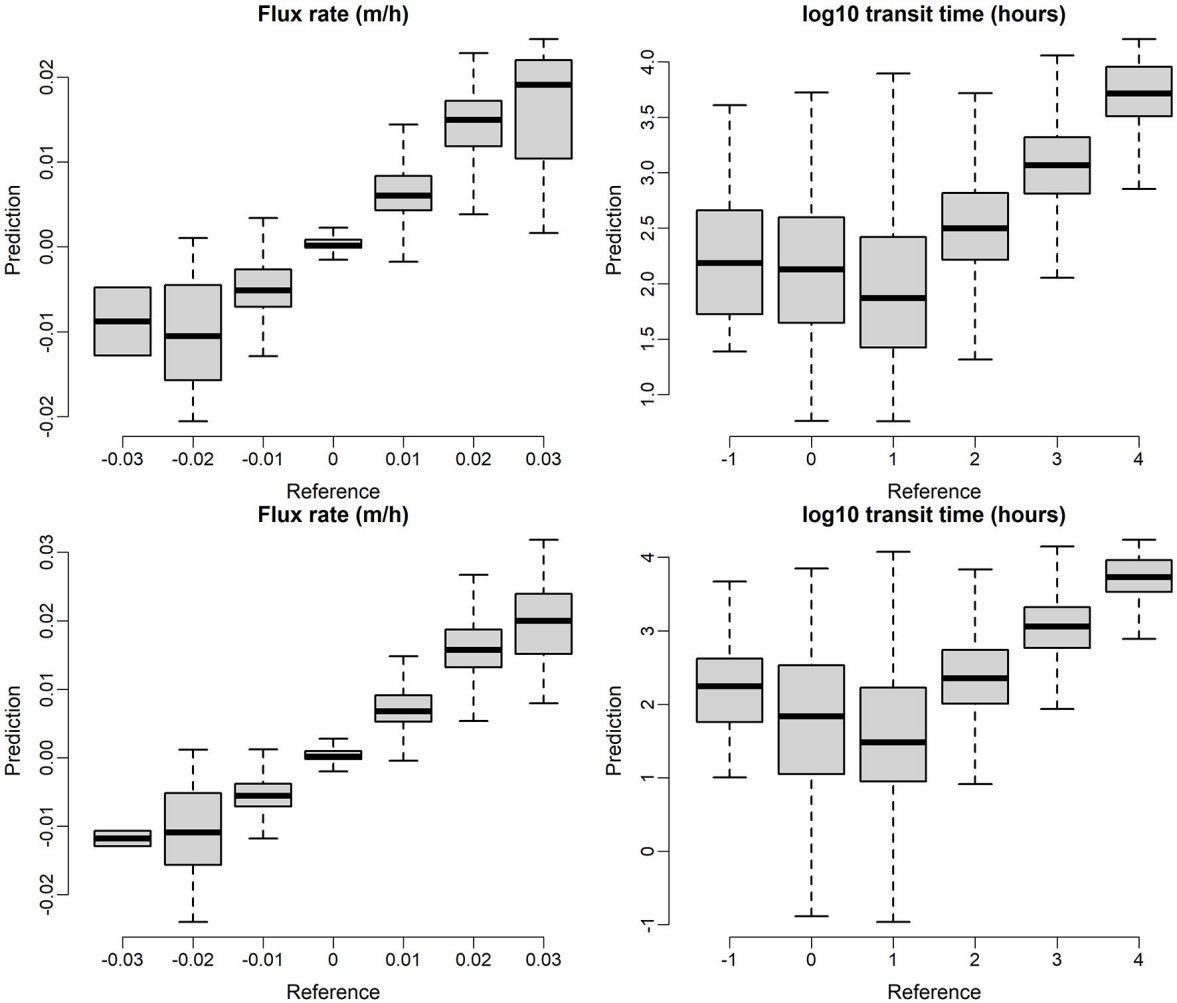
ML model prediction on testing dataset, the top panel is based on RF models and the bottom panel is based on XGB models.

Additional insight into the performance of model predictions can be obtained by additional comparison of model predictions to the testing data set. Here again we focus on the base configuration (C0) with all flows combined into a single data set. Figure 7 shows a comparison of model predictions vs. testing observations which the top panel is based on RF models and the bottom panel is based on XGB models. In both cases, data points are lumped into discrete categories and presented as box plots for visual simplicity. Perfect predictions would be represented by the centers of all box plots falling on a 1:1 line. We note that although XGB model is slight better than RF with relative narrower uncertainty bounds, the patterns in Figure 7 are very similar in general, indicating similar performance of the RF and XGB models for the base configuration. In general, the plots indicate good correspondence between model predictions and testing data. One notable exception is that both models struggle to accurately predict short transit times, with transit times being significantly overpredicted for the first two groupings for both RF and XGB. We can also observe that the flux is most accurately predicted when it is small (magnitude near zero).

### Alternative Input Data Configurations

Here we wish to explore whether alternative input data configurations (adding or removing input data variables) can improve the machine-learning model predictions. We consider two alternative data configurations to suit for different data availabilities and provide the guidance within the different ML models.

The first alternative configuration (C1) is the same as the base configuration with all flow periods combined into a single dataset, but with additional information added in the form of the spatial grid coordinates of each grid cell. This additional data may provide some information on spatial correlation or relationship between different grid cells that is not captured in the base configuration.

**Figure 8:**
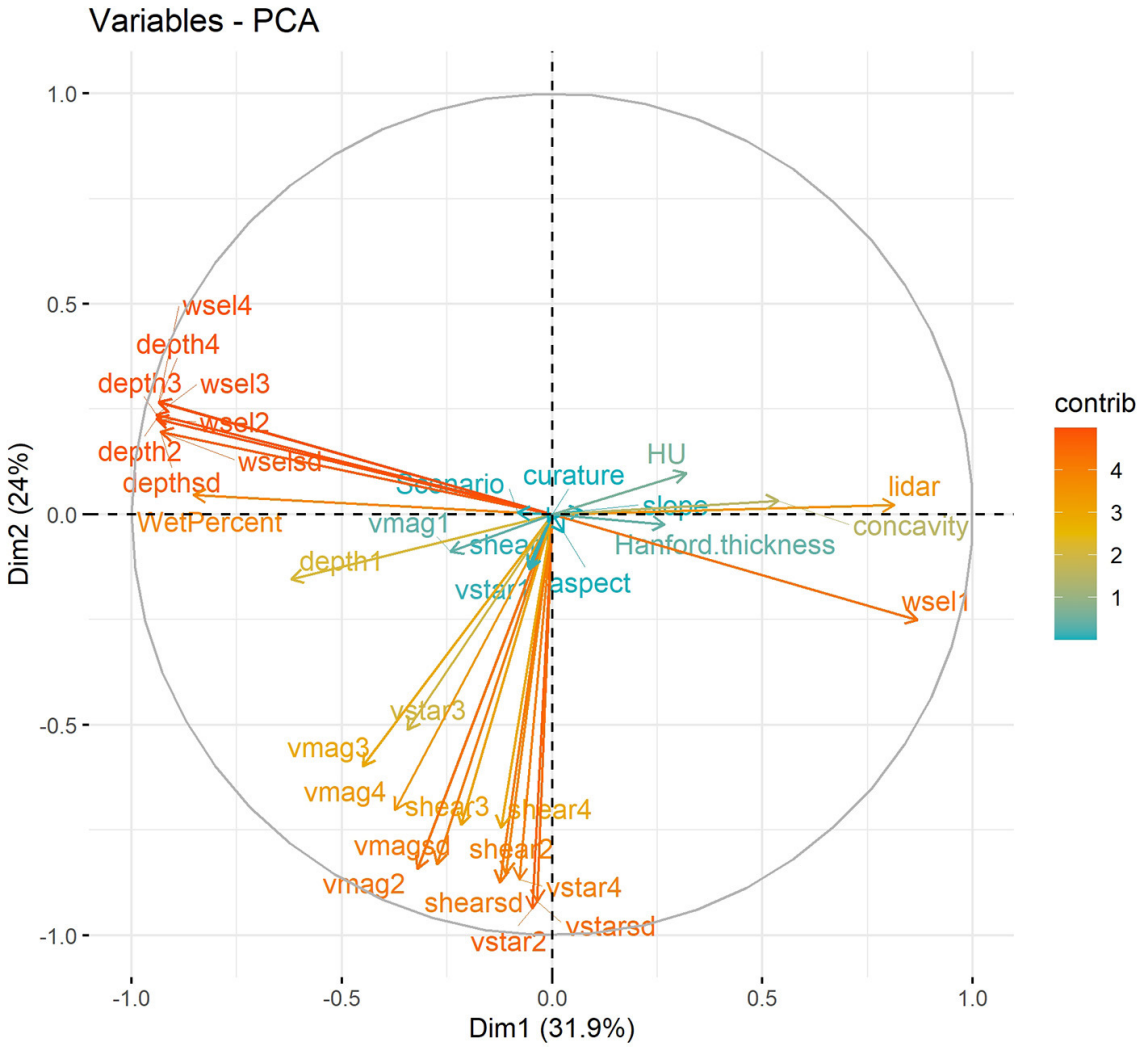
Results of PCA analysis for the predictor variables. Dim1 and Dim2 are the first- and second- identified principal components, respectively, and the arrows show the contributions of each predictor variable to those two principal components. Together, Dim1 and Dim2 capture over 55% of the total variance of the entire dataset.

For the second alternative configuration (C2), we performed PCA to identify sets of variables that were closely related (and therefore perhaps contained duplicative information). PCA analysis was performed using all predictors from the base configuration over the full time period. The results are shown in Figure 8, in which the horizontal and vertical axes show projections of the input variables onto the first- and second-principal components (PCs). Color of the arrows reflect the strength of the contributions of each variable to the first two PCs. From this figure we observe that water surface elevation, water depth, and their statistical moments primarily contribute to the second PC (Dim2). Elevation, HU type, and Hanford thickness can also be grouped based on their contributions to the second PC. The shear stress and velocity variables form a third group contributing strongly to the first PC (Dim1). Based on the outcome of the PCA analysis, we selected a reduced set of variables for inclusion in the input data for configuration C2. These variables are the flow regime, Hanford formation thickness, HU type, mean velocity magnitude, elevation, mean water surface elevation, mean bottom shear stress, mean shear velocity, mean water depth, and bathymetry slope. This list can be compared to, and is significantly reduced from, the list of variables for the base configuration as shown in Table 3.

**Figure 9:**
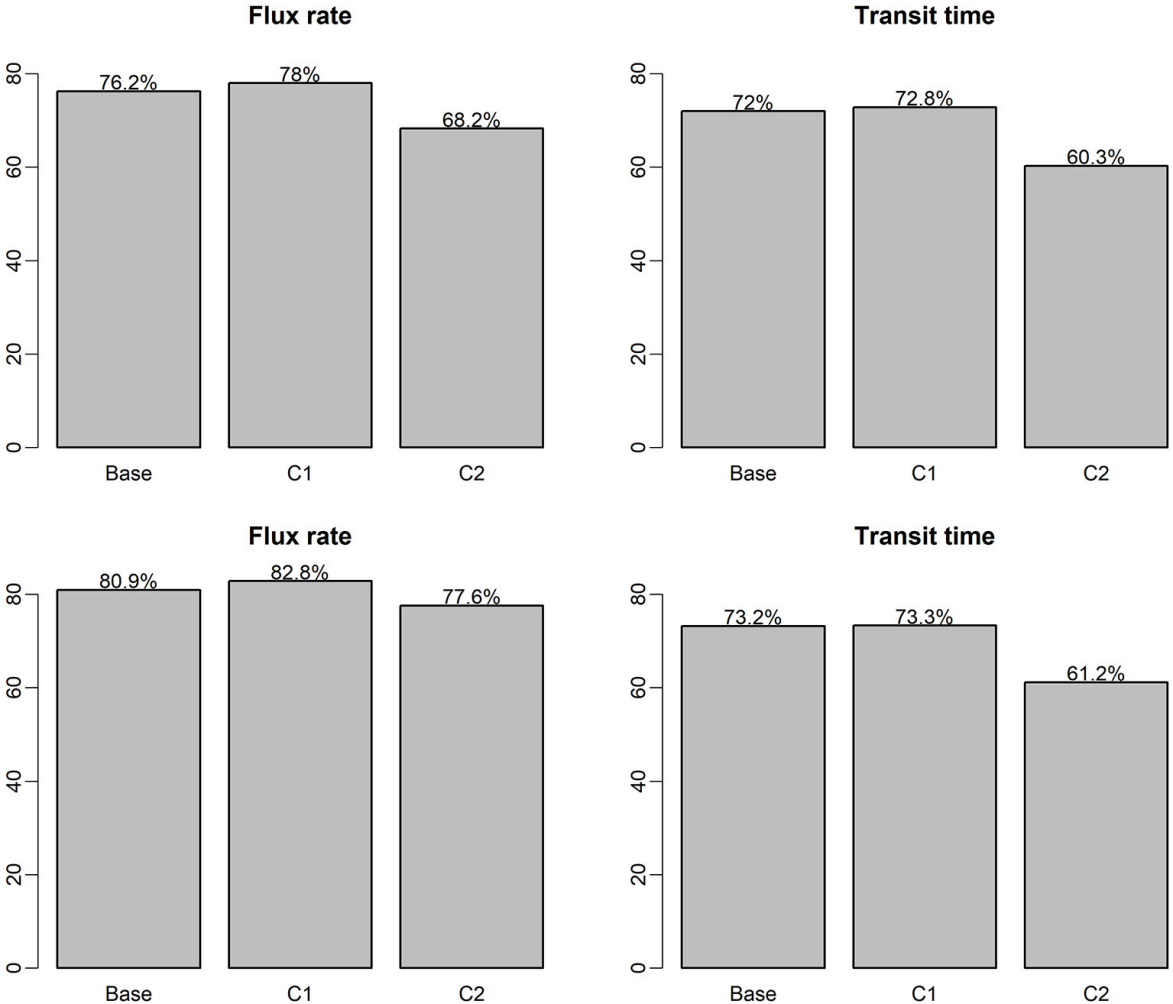
ML model *R*^2^ on testing dataset for different ML configurations, the top panel is based on RF models and the bottom panel is based on XGB models.

In comparison to the base case and C1, adding adjacent spatial coordinates information has limited improvement on model accuracy on both ML model (Figure 9). With RF and XGB model, about 2% improvement can be achieved on HEFs prediction and <1% improvement for TTDs prediction. This shows the developed model has the strong transferability with satisfied accuracy. For C2, the alternative configuration with reduced set of input variables, model accuracy for HEFs prediction only drops 8% and 3% for RF and XGB, respectively. To be specific, the XGB model still can achieve ~78% accuracy with reduced input variable set, which indicates the strong transferability of the model with limited data availability. In terms of the TTDs predictions, variable configuration C2, the accuracies are 60 and 61% for RF and XGM. It shows that the transit time is more complicated than exchange flux and may be impacted by multiple variables and their interactions.

### Impacts of Heterogeneous Riverbed Conductance

The impacts of heterogeneity in physical properties of riverbed and aquifer materials such as hydraulic conductivity have been previously evaluated in several studies (e.g., Tang et al., 2015; Stonedahl et al., 2018). The inferred importance of such heterogeneity can depend on the scale and character of the heterogeneity considered as well as the specific outcomes being predicted. Here we evaluated two cases, one with homogeneous riverbed conductance, and the second with heterogeneous conductance assigned based on identified hydromorphic feature associations.

**Figure 10:**
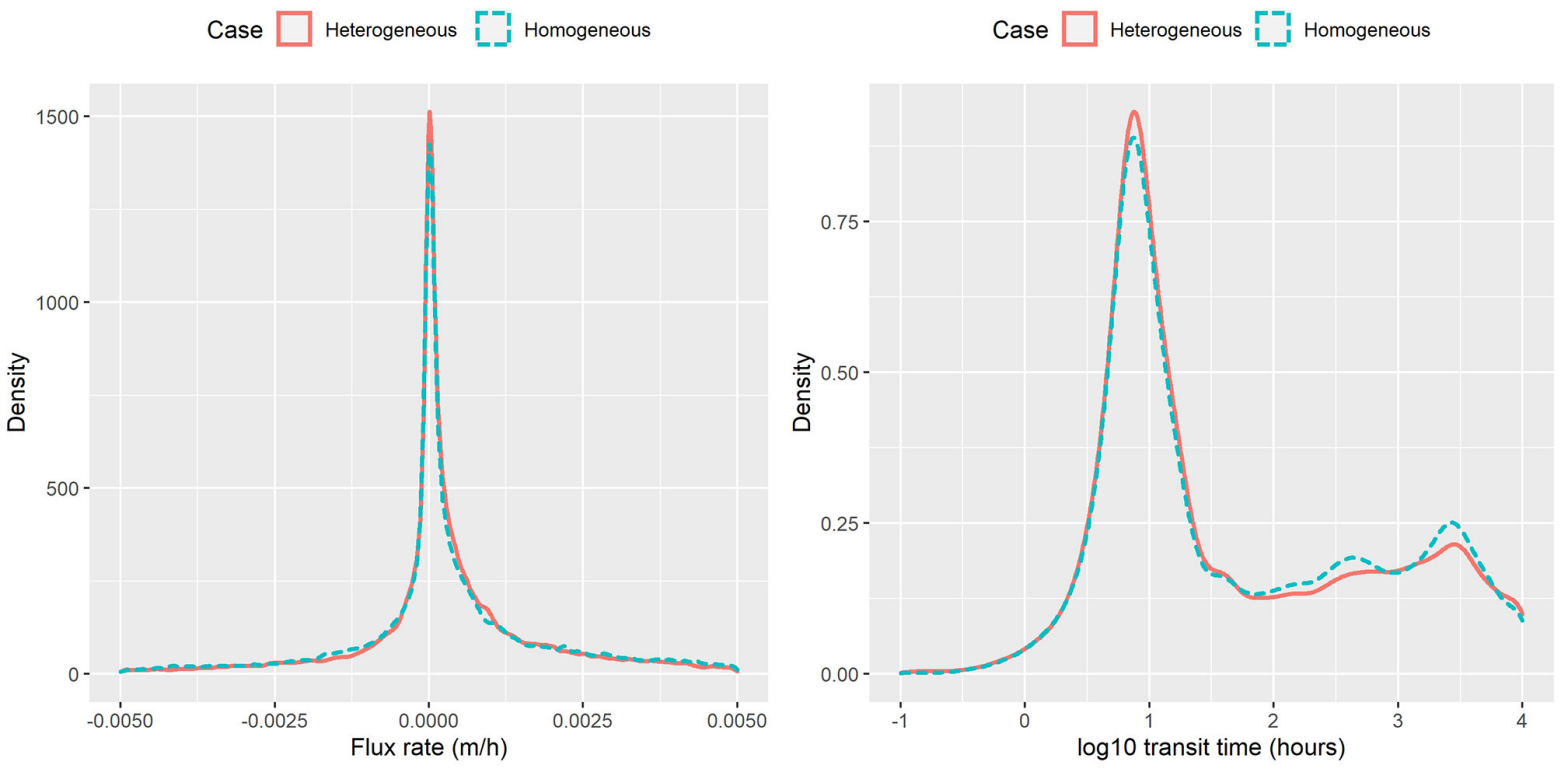
Flux rate and transit time obtained from particle tracking for homogeneous and heterogeneous cases.

The PDFs of exchange flux and transit time under the homogenous and heterogeneous cases in all flow regime are shown in Figure 10. In general, the overall density patterns are similar between the two cases, which is not surprising particularly because the derived HEFs and TTDs response variables were averaged behaviors of all particles, and such averaging smoothed out the finer scale differences due to spatial heterogeneity. The heterogeneous case tends to have slighter larger exchange fluxes and transit time compared with homogenous case. The impacts of the heterogeneity on HEFs and TTDs have been evaluated through our framework.

**Figure 11:**
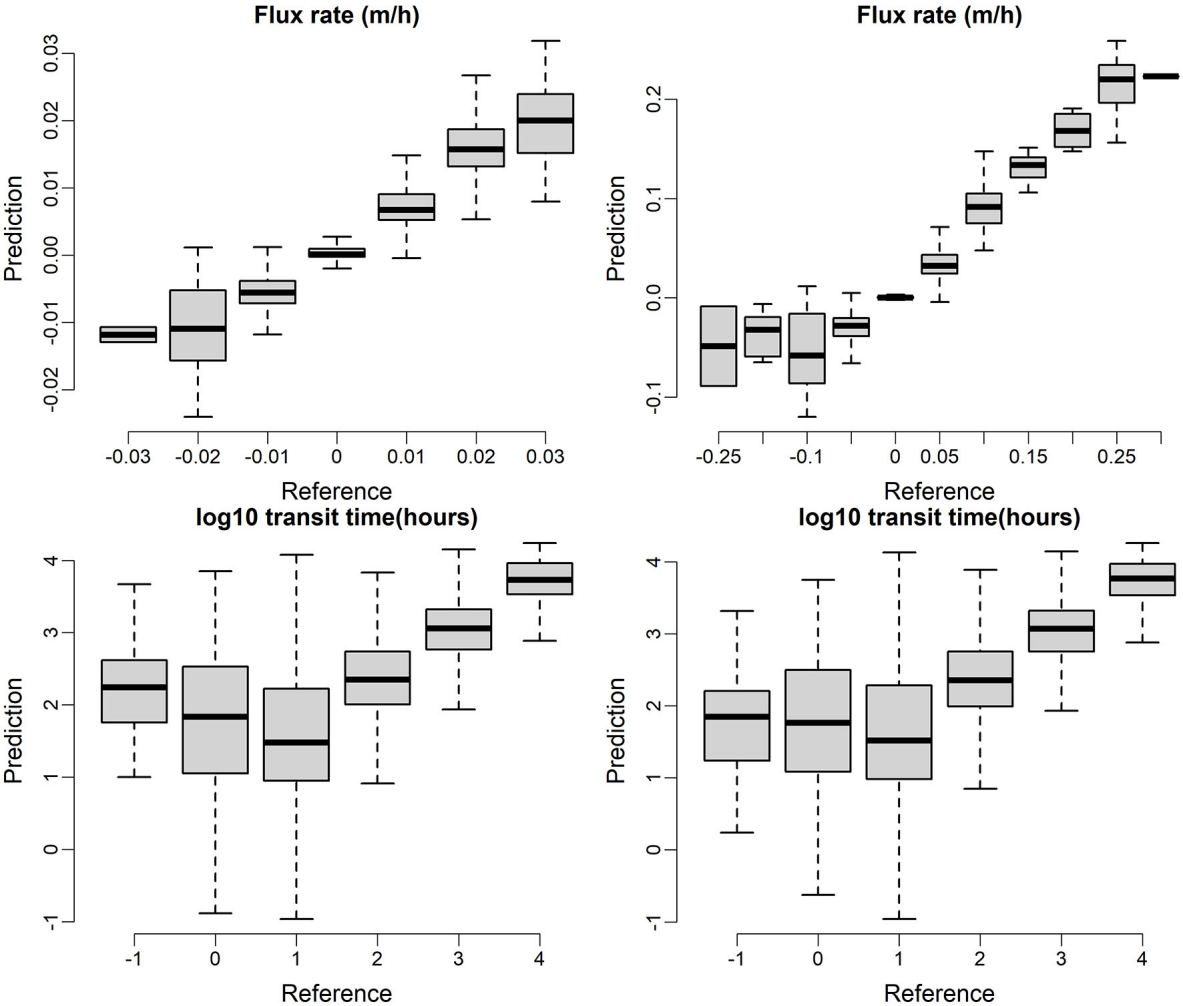
XGB model predictions for exchange flux (top) and transit time (bottom) on testing dataset for homogenous (left) and heterogenous (right) cases.

For homogeneous-heterogeneous, only XGB was considered (variable configurations C3 listed in Section Summary of Machine Learning Analyses Performed), and using base case and combined flow regimes (with categorical flow regime variable), because XGB beats RF on the various model setups. The XGB model predictions for both exchange flux and transit time on testing dataset are shown in Figure 11 where data points are lumped into bins. As shown in the top panel of Figure 11, good matches were found between model predictions and reference exchange fluxes for homogeneous-heterogeneous cases. Also, it is noticeable that the heterogeneous case enhanced the variability 10 times more than the homogenous case in exchange flux and the XGB model predictions are able to capture such large variability.

The transit time predictions are challenging for both the homogeneous and heterogeneous cases. For the transit times longer than 10 h, the predictions are accurate for both cases. However, for the short transit times, especially for the transit times less than 10 h, both homogeneous and heterogeneous predictions are significantly overestimated. It indicates that short term high frequency dynamic is more related to short transit time processes which are not reflected by the current ML model setup.

## Discussion and Conclusion

In this work, we adopted two ML approaches including RF and XGB for deriving the reduced order models for HEFs and TTDs along the Columbia River Corridor. Different model configurations have been used for comprehensive understanding of the influences of various factors such as spatial, temporal, geomorphologic and hydrodynamic attributes on exchange fluxes and transit times. Taking the advantage of the rich dataset available at the study site, variable importance has been ranked using different ML approaches. For the RF model, geomorphology and flow regime are the most influential factors for both HEFs and TTDs; while the hydrodynamic factors lead the significance for XGB models. The most influential predictors differ between the two ML models which indicates the importance of model selection depending on the data availability. Such data availability also attaches closely with model transferability to other river or larger study scales. In general, XGB performs better than RF, reaching 81 and 73% accuracy in predicting exchange flux and transit time respectively under the base configuration. The accuracies of models have been compared under alternative configurations of reduced set of input variables on both HEFs and TTDs to further demonstrate the model transferability.

Although many physical factors are known to exert control on HEFs and TTDs in coupled surface-subsurface flow, the development of a reduced-order or surrogate model requires variables that are easy to measure or compute at the corresponding study site. For example, field observations of local hydraulic gradients, a known primary controlling factor in hydrologic exchange, are typically very sparse and limited at most sites. Note that groundwater table elevations are relatively stable compared to river stage or discharge, therefore hydraulic gradient, which depends on the difference between river stage and groundwater table elevation, can be expected to be dominated by river stage fluctuations as a first-order approximation. Fortunately, river stage is a far more easily observed and simulated variable than hydraulic gradient, and is therefore more useful for surrogate model development. The dynamic pressure on the riverbed also depends on surface water velocity and riverbed topography, introducing dependencies on bathymetry and river discharge. We have selected input variables for our ML models that are relatively easily measured or modeled. We note that the ML validation tests reveal the extent to which the input variables under consideration (e.g., river stage, discharge, etc.) serve as reliable predictors of the desired outputs (HEFs and TTDs in this case), thus we can determine whether the variables we have chosen are sufficient or redundant for HEF/TTD inference. We note that our surrogate model is based on a fully connected river-aquifer system, and is not likely to be applicable to disconnected rivers with partially saturated zones beneath the riverbed (Schilling et al., 2017).

Compared with the physics-based numerical model, the wall-clock time for each ML model has been reduced to 1 ~ 2 h, depending on the number of trees built in the model, with 24 process cores on a supercluster. Note that the ML model development can be performed on local computer, where the large physics-based numerical model is not feasible.

From the domain perspective, good correspondence between model predictions and testing data has been observed for exchange fluxes, but both RF and XGB models have clearly overestimated the short transit times, because the transit time has more complex distribution patterns with strong temporal variability. It worth mentioning that the study site is a large-regulated river, where the frequent daily dam operations have impacted the short-transit-time high-frequency dynamics. Such short-term dynamics cannot seem to be captured by the current ML model setup. The temporal component in each flow regime-specific ML model has been evaluated as well to provide guidance on the suitable temporal resolution considering expensive numerical modeling and field measurements.

## Data Availability Statement

The raw data supporting the conclusions of this article will be made available by the authors, without undue reservation.

## Author Contributions

HR and ZH led the machine learning design and processed the model results, XS performed particle tracking, YF ran the 3D subsurface model and TS managed the overall contents and structure of the manuscript. All authors contributed to the article and approved the submitted version.

## Conflict of Interest

The authors declare that the research was conducted in the absence of any commercial or financial relationships that could be construed as a potential conflict of interest.
